# Supplementary material for: Metabolomic Response to Non-Steroidal Anti-Inflammatory Drugs
Source: Res Sq. 2024 Dec 9:rs.3.rs-5530702. Preprint. [Version 1] doi: 10.21203/rs.3.rs-5530702/v1 (PMC11661377; doi:10.21203/rs.3.rs-5530702/v1)
Supplement: Supplement 1 [file NIHPPRS5530702V1-supplement-1.pdf]

Supplementary Files

This is a list of supplementary files associated with this preprint. Click to download.

- SupplementaryTable1.docx
- Supplementary1.pdf
- Supplementary21.pdf
- Supplementary3.pdf
- Supplemenrtary4.pdf
- Supplementary5.pdf
- Supplementay6.pdf
- Supplementary7.pdf
- Supplementary8.pdf
- Supplementary9.pdf
- Supplementary10.pdf
- SupplementaryFigurelegends.docx
